# 3’UTR of SARS-CoV-2 spike gene hijack host miR-296 or miR-520h to disturb cell proliferation and cytokine signaling

**DOI:** 10.3389/fimmu.2022.924667

**Published:** 2022-09-27

**Authors:** Jinjin Yuan, Ziheng Feng, Qiaowen Wang, Lifen Han, Shenchan Guan, Lijuan Liu, Hanhui Ye, Lili Xu, Xiao Han

**Affiliations:** ^1^ Department of Infectious Diseases, Mengchao Hepatobiliary Hospital of Fujian Medical University, Fuzhou, China; ^2^ Beijing Key Laboratory of Pediatric Respiratory Infection Diseases, Key Laboratory of Major Diseases in Children, Ministry of Education, National Clinical Research Center for Respiratory Diseases, National Key Discipline of Pediatrics (Capital Medical University), Beijing Pediatric Research Institute, Beijing Children’s Hospital, Capital Medical University, National Center for Children’s Health, Beijing, China; ^3^ College of Biological Science and Engineering, Fuzhou University, Fuzhou, China

**Keywords:** SARS-CoV-2, cell proliferation, 3’UTR, cytokine signaling, spike gene

## Abstract

Severe acute respiratory syndrome coronavirus 2 (SARS-CoV-2) has becoming globally public health threat. Recently studies were focus on SARS-CoV-2 RNA to design vaccine and drugs. It was demonstrated that virus RNA could play as sponge to host noncoding RNAs to regulate cellular processes. Bioinformatic research predicted a series of motif on SARS-CoV-2 genome where are targets of human miRNAs. In this study, we used dual-luciferase reporter assays to validate the interaction between 3’UTR of SARS-CoV-2 S (S-3’UTR) gene and bioinformatic predicted targeting miRNAs. The growth of 293T cells and HUVECs with overexpressed S-3’UTR was determined, while miRNAs and IL6, TNF-α levels were checked in this condition. Then, miR-296 and miR-602 mimic were introduced into 293T cells and HUVECs with overexpressed S-3’UTR, respectively, to reveal the underlying regulation mechanism. In results, we screened 19 miRNAs targeting the S-3’UTR, including miR-296 and miR-602. In 293T cell, S-3’UTR could inhibit 293T cell growth through down-regulation of miR-296. By reducing miR-602, S-3’UTR could induce HUVECs cell proliferation, alter the cell cycle, reduce apoptosis, and enhanced IL6 and TNF-αlevel. In conclusion, SARS-CoV-2 RNA could play as sponge of host miRNA to disturb cell growth and cytokine signaling. It suggests an important clue for designing COVID-19 drug and vaccine.

## Introduction

Since the appearance of coronavirus disease 2019 (COVID-19) in December 2019, the disease has spread globally, becoming the most significant public health threat in the world today. COVID-19 is a novel coronavirus disease caused by severe acute respiratory syndrome coronavirus 2 (SARS-CoV-2). Currently, millions of people have been diagnosed with SARS-CoV-2 worldwide (https://covid19.who.int) and the growing number of individuals with COVID-19 has placed a burden on the healthcare systems of many countries. However, to date, no effective antiviral drugs have been approved to treat COVID-19. The most common initial symptoms of COVID-19 are fever and respiratory symptoms such as cough, shortness of breath, and sore throat. Furthermore, COVID-19 can also affect the heart and blood vessels, promoting the development of cardiovascular diseases such as myocardial damage, arrhythmia, acute coronary syndrome (ACS), and venous thromboembolism. At present, the pathogenic mechanism of the new coronavirus epidemic has not fully been elucidated and most people are still at risk of contracting COVID-19. Further exploration of the pathogenic mechanism of COVID-19 is therefore essential to control and treat this disease.

miRNA plays an important regulatory role in many biological processes and is instrumental in the interaction between viruses and hosts. Host miRNA can be used as a “weapon” to interfere with virus replication. Conversely, viruses can regulate host miRNA to suppress the host immune system. Previous studies have identified differentially expressed miRNAs in COVID-19 patients through transcriptome analysis (1–5). In particular, virus RNA can play as sponge of host miRNA to regulate immune processes. It was described hepatitis C virus RNA sequesters host miR-122 to facilitate viral replication (6). Recent study revealed the potential miRNA interacted with SARS genomes in human cell (7). In another study, it was demonstrated endogenous human micro and long non-coding RNAs has potential binding site in the SARS-CoV-2 genome (8). These evidences are clue for RNA-base drugs and understanding pathogen mechanism of COVID-19. However, no experimental evidence proofed that SARS-CoV-2 RNA could play as sponge of host miRNA involved in COVID-19 disease.

Here, the current study screens some of the miRNAs that 3’UTR of SARS-CoV-2 S gene can target and further investigates whether 3’UTR of SARS-CoV-2 S gene can affect cell proliferation and expression levels of cytokines through sponging to these miRNAs.

## Results

### Screening for miRNAs targeting the 3’UTR of SARS-CoV-2 S gene

**Figure 1:**
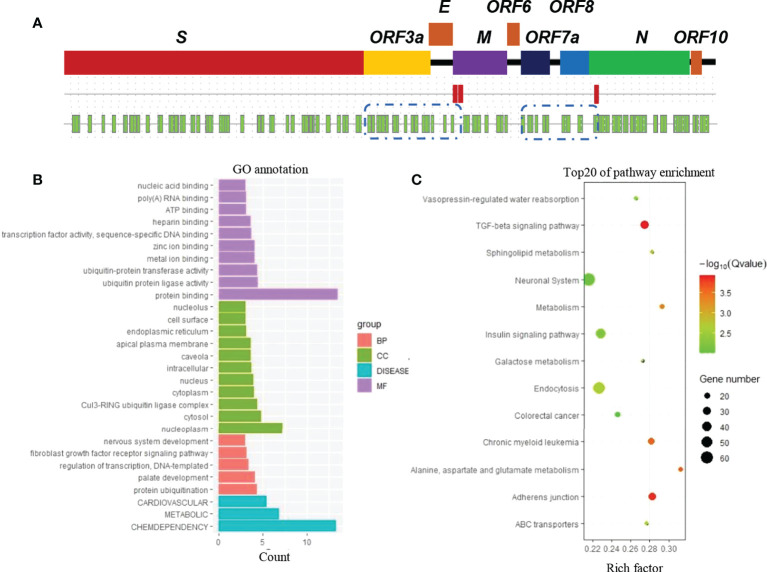
Prediction of human miRNAs binding to SARS-CoV-2 RNA. **(A)** Genome map of SARS-CoV-2 (first line), potential PolyA sites (red block in the second line), and potential miRNA binding sites (green blocks in the third line). **(B)** Gene classification. Genes are the predicted targets of miRNAs that have a predicted interaction with SARS-CoV-2 RNA. **(C)** Kyoto Encyclopedia of Genes and Genomes (KEGG) enrichment analysis.

In the SARS-CoV-2 genome, two potential PolyA sites were identified after the *S* gene by using bioinformatics web tools (http://regrna2.mbc.nctu.edu.tw/) (**Figure 1A**). The polyA site furthest from the stop code of the *S* gene was used to define the 3′-untranslated region (3′UTR). Subsequently, 19 miRNAs with a predicted interaction site on the 3′UTR of the *S* gene (S-3′UTR) were identified by screening a bioinformatic database (**Figure 1A**; **Supplemental Table 1**).

Potential target genes of the 19 miRNAs were also predicted using the same database. According to Gene Ontology (GO) annotation, these target genes are involved in cardiovascular, metabolic, and chemdependency disease pathways (**Figure 1B**). Furthermore, these genes were suggested to be enriched in biological pathways such as TGF-beta signaling, adherens junction, and metabolism (**Figure 1C**).

**Figure 2:**
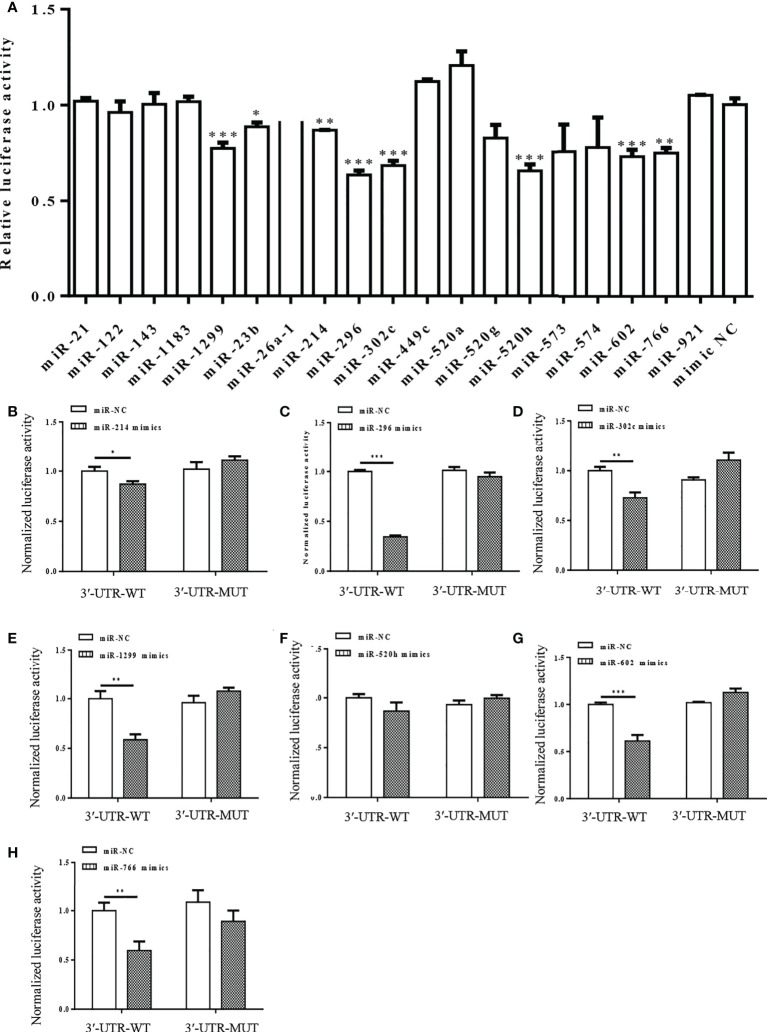
Dual-luciferase reporter assay showing interaction between human miRNAs and S-3′UTR of SARS-CoV-2. **(A)** Luciferase activity was detected 48 h after co-transfection of cells with S-3′UTR-WT reporter and miR-NC or miRNA mimics. **(B-H)** Normalized Luciferase activity was detected 48 h after co-transfection of cells with S-3′UTR-WT and S-3′UTR-MUTANT reporter and miR-NC or miRNA mimics. miRNAs include miR-214 **(B)**, miR-296 **(C)**, miR-302c **(D)**, miR-1299 **(E)**, miR-520h **(F)**, miR-602 **(G)**, and miR-766 **(H)**. (*p < 0.05, **p < 0.01, ***p < 0.001).

Interactions between the miRNAs and S-3′UTR were validated *via* a series of dual-luciferase reporter assays in 293T cells. Eight miRNAs significantly reduced the S-3′UTR reporter signal, including miR-1299, miR-23b, miR-214, miR-296, miR-302c, miR-520h, miR-602, and miR-766. Luciferase activities were decreased by about 20%–50% in the miRNA mimics group compared with the control (NC) group (**Figure 2A**).

Mutant S-3′UTRs were generated to disrupt potential interaction sites between miRNAs and S-3′UTR. Dual-luciferase reporter assays were repeated with mutant S-3′UTR controls and revealed that miR-1299, miR-23b, miR-214, miR-296, miR-302c, miR-520h, miR-602, and miR-766 could reduce the S-3′UTR reporter signal but not that of the mutant S-3′UTR (**Figures 2B-H**). Among these miRNAs, miR-296 caused the largest reduction in the reporter signal (**Figure 2C**).

### S-3′UTR overexpression downregulated miR-296 and inhibited growth of 293T cells

**Figure 3:**
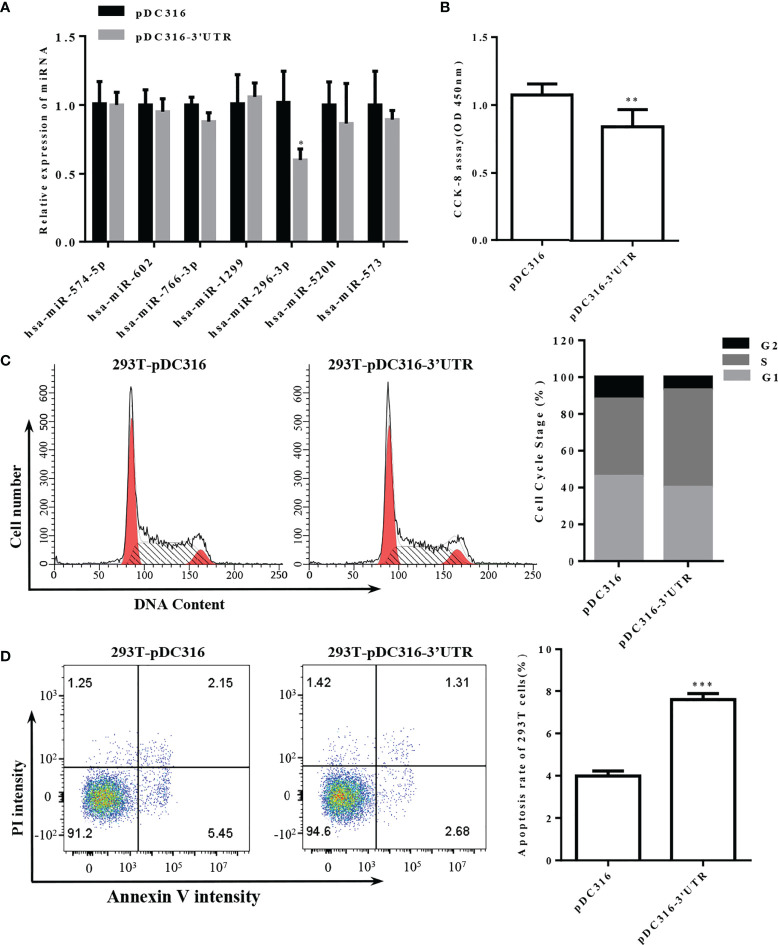
Overexpression of S-3′UTR downregulated miR-296 expression and disturbed 293T cell growth. **(A)** Expression levels of miRNAs following overexpression of S-3′UTR in 293T cells. Note that only expression of miR-296 was reduced. **(B)** 293T cell proliferation was detected by CCK-8. Note that overexpression of S-3′UTR decreased cell proliferation. **(C)** Distribution of 293T cell cycle. Note that overexpression of S-3′UTR increased the proportion of cells in S phase. **(D)** Apoptotic rate was detected by flow cytometry in 293T cells. Overexpression of S-3′UTR increased apoptosis of 293T cells. (*p < 0.05, **p < 0.01,***p < 0.001).

To study the biological cellular function of SARS-CoV-2 S-3′UTR, a vector was constructed to overexpress this region in 293T cells. The expression levels of seven miRNAs that possess the ability to bind S-3′UTR were then determined. Only miR-296-3p was significantly downregulated in 293T cells after overexpression of S-3′UTR, while expression of the other six miRNAs did not change significantly (**Figure 3A**).

The proliferation ability of 293T cells overexpressing S-3′UTR was checked by CCK-8 assay and was significantly reduced by approximately 33% compared with the control (**Figure 3B**). Cell-cycle monitoring revealed that the number of cells in S phase was increased by approximately 10% in the S-3′UTR group compared with the control group (**Figure 3C**). Flow cytometry demonstrated that the amount of apoptosis in the S-3′UTR group was twice that of the control group (**Figure 3D**).

### Overexpression of miR-296 could recover the 293T cells disturbed by S-3’UTR

**Figure 4:**
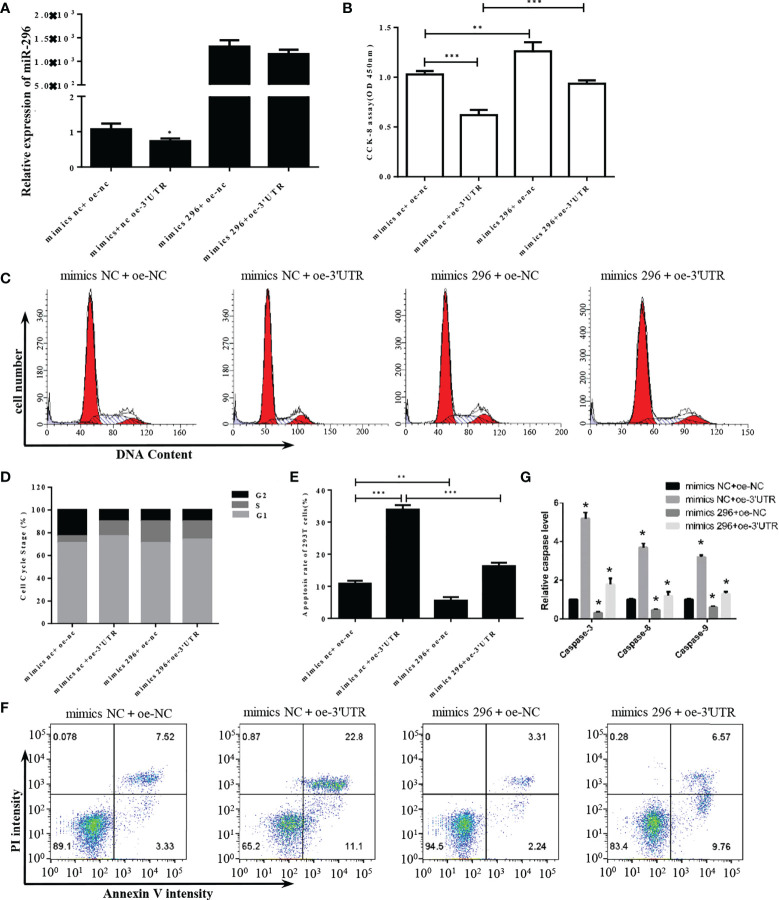
S-3′UTR disturbed 293T cell growth though knock-down of miR-296. **(A)** Level of miR-296 expression in 293T cells. **(B)** Reduced 293T cell proliferation induced by overexpression of S-3′UTR was partially recovered by miR-296 mimics. **(C, D)** Effects of S-3′UTR on 293T cell cycle could not be recovered by miR-296 mimics. **(E, F)** Apoptosis of 293T cells induced by overexpression of S-3′UTR was partially reduced by miR-296 mimics. **(G)** caspase-3/8/9 assay to check apoptosis. (*p < 0.05, **p < 0.01, ***p < 0.001).

To further explore the functional mechanism of S-3′UTR through miR-296, a microRNA mimic was used to restore the level of miR-296 in 293T cells overexpressing S-3′UTR (**Figure 4A**). Cell proliferation in the S-3′UTR group without microRNA mimic was significantly reduced by approximately 40% compared with the control, while cells overexpressing S-3′UTR plus miR-296 mimic exhibited restored proliferation ability, at approximately 90% of the control cells (**Figure 4B**). In addition, the miR-296 mimic group without S-3′UTR increased the cell proliferation ability by 20%. Cell-cycle analysis revealed that the S-3′UTR, miR-296 mimic, and S-3′UTR plus miR-296 mimic groups all had an increased percentage of cells in S phase and a decreased percentage of cells in G2 phase compared with the control (**Figures 4C, D**). Furthermore, the S-3′UTR group showed an increased apoptosis ratio of three times that of the control group, while the S-3′UTR plus miR-296 mimic group only showed an increase of 1.5 times that of the control group. In addition, the miR-296 mimic group exhibited a 50% reduction in apoptosis ratio compared with control (**Figures 4E, F**). The caspase assay checked the level of caspase-3/8/9, results indicated the same pattern of apoptosis (**Figure 4G**). This suggested that miR-296 could recover the cell proliferation and reduce apoptosis in 293T cells disturbed by S-3′UTR.

### S-3’UTR decreased expression of miRNAs, increased cell proliferation, altered the cell cycle, reduced apoptosis, and enhanced cytokine expression in HUVECs

**Figure 5:**
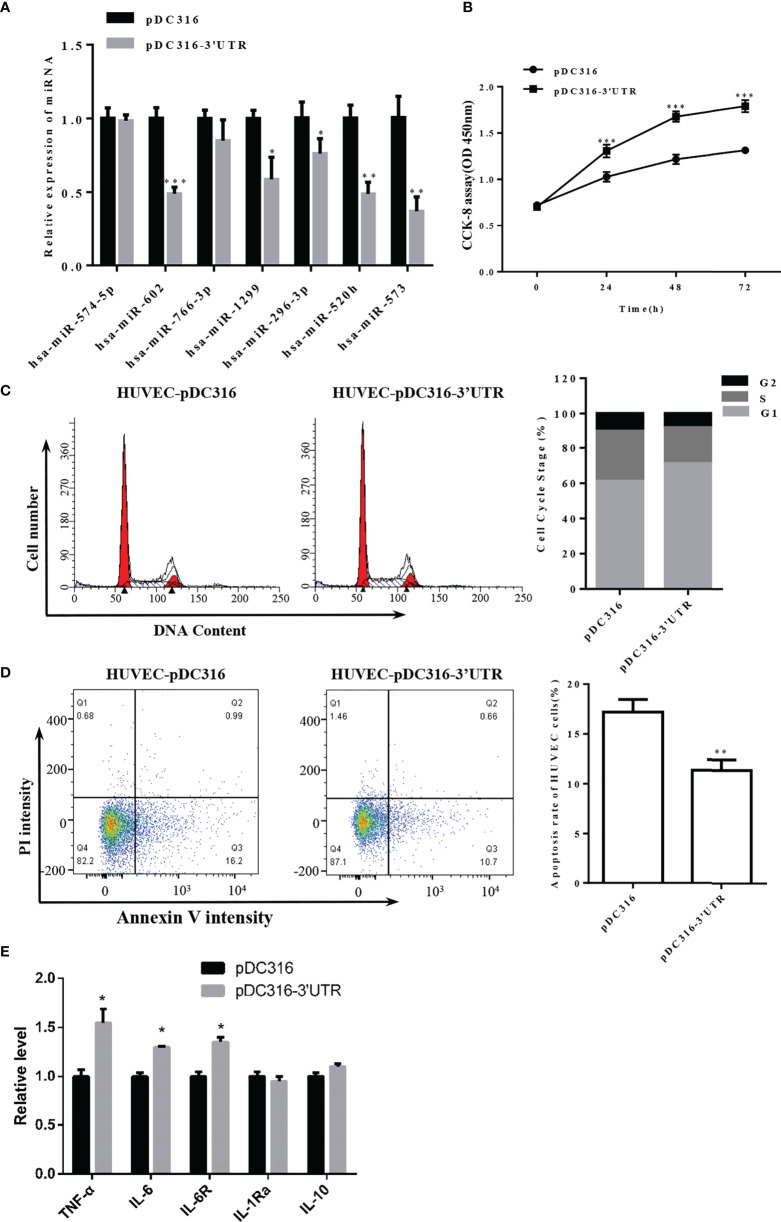
Overexpression of S-3′UTR downregulated miR-296 and disturbed 293T cell growth. **(A)** Expression levels of miRNAs following overexpression of S-3′UTR in HUVECs. Note that miR-602 was the most significant. **(B)** Overexpression of S-3′UTR enhanced HUVEC proliferation. **(C)** Overexpression of S-3′UTR increased the proportion HUVECs in G1 phase. **(D)** HUVEC apoptotic rate was reduced by overexpression of S-3′UTR. **(E)** Level of IL-6, IL-6R and TNF-α was increased following overexpression of S-3′UTR. (*p < 0.05, **p < 0.01).

SARS-CoV-2 can directly target endothelial cells. Therefore, to further explore the effect of S-3′UTR on endothelial cells, a viral vector was constructed to overexpress S-3′UTR in HUVECs. Expression levels of miRNAs and cell phenotypes were then determined in these cells. Among seven miRNAs targeted to S-3′UTR, expression of miR-602, miR-1299, miR-296, miR-520h, and miR-573 was significantly reduced in HUVECs overexpressing S-3′UTR (**Figure 5A**).

Cell proliferation ability of HUVECs in the S-3’UTR group was increased by approximately 40% compared with the control group (**Figure 5B**). Furthermore, the proportion of cells in G1 phase increased approximately 10% in the S-3′UTR group (**Figure 5C**), and the percentage of apoptotic cells in the S-3’UTR group was reduced to 10%, whereas it was approximately 17% in the control (**Figure 5D**). Moreover, levels of cell secreted IL-6 and TNF-α and IL-6R in cell—important molecules in the cytokine storm caused by SARS-CoV-2 infection—were determined. IL-6 and TNF-α were significantly upregulated, by approximately 12% and 40%, respectively, in S-3′UTR group compared with the control group, IL-6R were also increased in cells, other anti-inflammatory cytokines such as IL-10 and IL-1Ra has no significant difference (**Figure 5E**).

### Overexpression of miR-520h could recover the HUVECs disturbed by S-3′UTR

The role of miR-S-3’UTR interaction(s) was further clarified by analyzing potential target genes of miRNAs that were downregulated by S-3′UTR in HUEVCs. Among miR-602, miR-1299, miR-296, miR-520h, and miR-573, only miR-520h has predicted gene targets, which are IL-6R and HiF-α, molecules involved in IL-6 and TNF-α signaling pathways.

**Figure 6:**
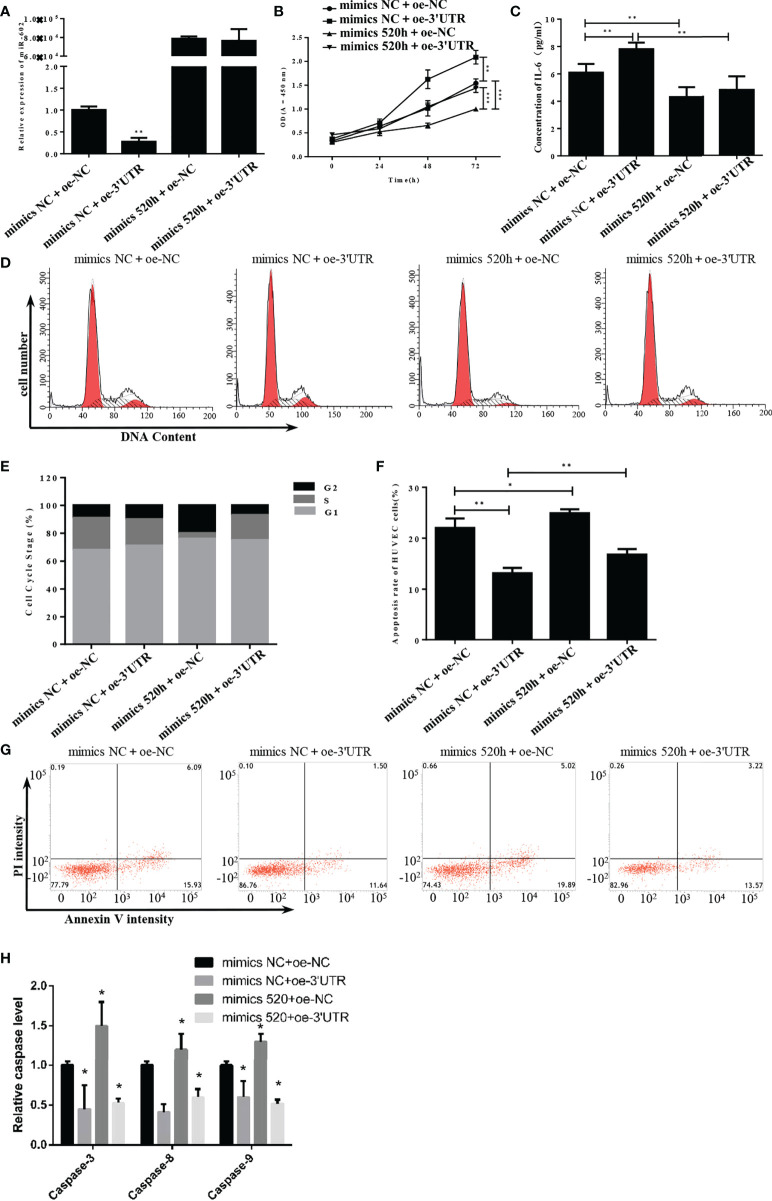
S-3′UTR disturbed growth of HUVECs by knock-down of miR-520h. **(A)** Level of miR-520h in HUVECs. **(B)** HUVEC proliferation induced by overexpression of S-3′UTR was reduced by miR-520h mimics. **(C)** Increased IL-6 level induced by overexpression S-3′UTR was reduced by miR-520h mimics. **(D-G)** Effects of S-3′UTR on HUVECs cell cycle could not be recovered by miR-520h mimics. **(E, F)** Apoptosis of HUVECs induced by overexpression of S-3′UTR was partially reduced by miR-520h mimics. **(H)** caspase-3/8/9 assay to check apoptosis. (*p < 0.05, **p < 0.01, ***p < 0.001).

Analysis of miR-520h expression revealed that miR-520h was significantly downregulated following overexpression of 3′UTR but markedly upregulated after transfection with the miR-520h mimic (**Figure 6A**). Accordingly, S-3′UTR significantly induced the cell proliferation ability and the miR-520h mimic reduced the cell proliferation ability by approximately 30%, while the S-3’UTR plus miR-520h mimic restored the cell proliferation ability to that of the control group (**Figure 6B**). Next, IL-6 concentrations were examined using an enzyme-linked immunosorbent assay (ELISA). IL-6 was significantly increased in the S-3′UTR group and restored to the control level in the S-3′UTR plus miR-520h mimic group (**Figure 6C**). Analysis of cell cycle distribution showed that the miR-520h mimic group had a markedly reduced percentage of cells in S phase and an increased number of cells in G2 phase. In contrast, the S-3’UTR group and S-3’UTR plus miR-520h mimic group had an increased number of cells in G1 phase (**Figures 6D, E**). Furthermore, the amount of apoptosis in the S-3′UTR group was reduced to 12.5%, while the S-3′UTR plus miR-520h mimic group had 17.5% apoptotic cells (**Figures 6F, G**). The caspase assay checked the level of caspase-3/8/9, results indicated the same pattern of apoptosis (**Figure 6H**). These observations indicate that miR-520h could recover the HUVECs disturbed by S-3′UTR.

## Discussion

SARS-CoV2 predominantly infects nasal and bronchial epithelial cells, type I and type II alveolar pneumocytes, and capillary endothelial cells *via* the viral structural spike (S) protein binding to the ACE2 receptor (9). In responding to infection, T lymphocytes, monocytes, and neutrophils are recruited to infection sites and secrete various cytokines, such as TNF-α, IL-1, and IL-6. During the late stage of infection, hyper-inflammation—also known as a “cytokine storm”—results in alveolar interstitial thickening, pulmonary edema, and compromised epithelial-endothelial barrier integrity, collectively referred to as acute respiratory distress syndrome (ARDS) (10). ARDS and multiorgan dysfunction are two leading causes of death in severe cases of COVID-19 and are frequent consequences of cytokine storm (11). Cytokines are an essential part of the inflammatory response, participating in a wide range of pathophysiological processes and assisting the host in eliminating pathogens. However, inappropriate inflammatory responses trigger over-production of cytokines, which causes tissue damage. Cytokine storm is an umbrella term describing a group of clinical manifestations caused by cytokine dysregulation, such as systemic inflammation, constitutional symptoms, and multiorgan dysfunction (12). An aggressive inflammatory response, accompanied by high levels of chemokines and cytokines, such as IL-2, IL-6, IL-7, IL-10, TNF-α, IFN-γ, IP10, and MCP1, was identified in patients with COVID-19 (13–15). Patients requiring admission to intensive care units (ICU) displayed higher levels of proinflammatory cytokines including IL-6 and TNF-α (13, 15).

IL-6 is a cytokine that promotes inflammation, immune reactions, and hematopoiesis (16), while TNF-α plays an important role in cellular activation and recruitment of leukocytes to inflammatory sites (17). IL-6 and TNF-α are two crucial cytokines in the pathogenesis of the SARS-CoV-2-induced cytokine storm and are probably responsible for severe clinical presentation and poor prognosis (18). In the current study, overexpression of S-3′UTR was observed to significantly upregulate both IL-6 and TNF-α in HUVEC cultures. The interaction between miR-520h and S-3′UTR was likely responsible for this upregulation since overexpression of S-3′UTR in HUVECs led to a significant decrease in expression of miR-520h and this could be restored by inclusion of a miR-520h mimic. Bioinformatic analysis identified that miR-520h could downregulate IL-6R and HiF-α and might play a role in regulating immune homeostasis and inflammation during SARS-CoV-2 infection. IL-6 binds membrane-bound IL-6R (mIL-6R), which induces homodimerization of membrane-bound gp130 (mgp130), or alternatively, IL-6 can bind to soluble IL-6R (sIL-6R), forming a complex of IL-6 and sIL-6R to interact with mgp130, and then initiates downstream signaling cascades (19). HiF-α is an important signaling molecule contributing to pathological and physiological changes of homoeostasis under hypoxia stress (20). Downregulation of HiF-α was previously shown to inhibit the expression of IL-6 and TNF-α (21). We hypothesize that miR-520h was targeted by S-3′UTR and thus the biological functions of miR-520h—including inhibition of IL-6R and HiF-α—were silenced, leading to inappropriate secretion of IL-6 and TNF-α and a severe inflammatory response.

Changes in the expression levels of miRNAs identified by transcriptomic and bioinformatic analysis in SARS-CoV-2 infected patients have been reported and include the upregulation of miR-2392 and miR-3605-3p, and the downregulation of miR-146a-5p, miR-21-5p, and miR-142-3p (22, 23). Furthermore, differentially expressed genes in SARS-CoV-2-infected human lung epithelial cells were predicted to be targeted by some miRNAs, such as hsa-miR-342-5p, hsa-miR-432-5p, hsa-miR-98-5p, and hsa-miR-17-5p (24). These studies indicate that miRNAs have a role in the pathogenesis of SARS-CoV-2. miRNAs are a group of single-stranded, small (~21–22 nt), noncoding RNAs, which are known as negative regulators of gene expression and essential biological processes predominantly through binding to the 3′UTR of target mRNAs at post-transcriptional levels (25). miRNAs can bind to a wide range of RNA viruses, mostly in the 5′- and 3′-UTRs and regulate viral pathogenesis in different ways (26, 27). For example, miR-323, miR-491, and miR-654 could bind to the PB1 gene of H1N1 influenza A virus and subsequently suppress its replication (28), while miR-28-5p, miR-150, miR-223, and miR-382 could decrease HIV-1 translation but were pivotal in HIV-1 latency and reservoir (29). In the current study, 19 miRNAs with a predicted interaction site on the 3′UTR of the *S* gene of SARS-Cov-2 were identified by screening a bioinformatic database and eight of these miRNAs were subsequently proven to bind to S-3′UTR in a series of dual-luciferase reporter assays. These eight miRNAs were miR-1299, miR-23b, miR-214, miR-296, miR-302c, miR-520h, miR-602, and miR-766. In addition to miR-602 described above, miR-766, miR-214, miR-23b, miR-1299, miR-520h, and miR-302 were previously reported to be highly expressed in tumor cells that were frequently involved in the processes of carcinogenesis, tumor progression, and metastasis (30–35). Expression levels of miR-296 were frequently associated with cardiovascular diseases. In the current study, they could directly bind to the S-3′UTR of SARS-CoV-2, while their mutant could not reduce the signal of S-3′UTR. In addition, RNA viruses can induce changes in cellular miRNA expression, which might help the viruses replicate and avoid host immune responses. Influenza virus could downregulate miR-24 levels in A549 cells to increase expression of furin protein and allow the progeny virus numbers to increase (36). Coronavirus OC43 nucleocapsid protein downregulated expression of miR-9, which is a negative regulator of NF-κB, leading to continual NF-κB translation (37). Decreased levels of miR-221 were found in RSV-infected human bronchial epithelial cells, and this could prevent cell apoptosis and boost viral replication (38). In the present study, miR-296 was significantly downregulated after overexpression of S-3′UTR in 293T cells, while miR-602, miR-1299, miR-296, miR-520h, and miR-573 were significantly downregulated after overexpression of S-3′UTR in HUVECs. In 293T cells overexpressing S-3′UTR, only miR-296-3p was significantly downregulated. Furthermore, cell proliferation ability was suppressed approximately 33% compared with the control, and from the cell-cycle analysis, more cells (approximately 10% increase compared with control) were trapped in S phase. miR-296 could regulate cellular proliferation *via* several pathways.

First, the p53/p21 axis is a crucial mediator in cell cycle control. Yoon et al. showed that overexpression of miR-296 could suppress the p53-p21 pathway and decrease mRNA expression of p21 by targeting the 3′UTR of p21 (39). miR-296-3p could also target phosphatase and tension homologue (PTEN) and inhibit its downstream phosphoinositide 3-kinase (PI3K)/Akt signaling pathway (40), which is involved in cell proliferation, growth, cell size, metabolism, and motility (41). S-3′UTR of SARS-CoV-2 probably disturbed the physiological function of miR-296 and subsequently led to suppression of proliferation. In addition, the S-3′UTR group displayed enhanced apoptosis activities compared with the control group. In HUVECs overexpressing S-3′UTR, downregulation of miR-520h led to significant induction of cell proliferation and reduction of apoptosis activity. The profile of the cell cycle also changed, with the proportion of cells in G1 phase increasing by approximately 10%. miR-520h has a negative effect on HUVECs cell proliferation and other tumor cells including granulosa-like tumor cell (35, 42–45). It was reported that miR-520h could target on IL-6R to inhibit cell growth (42).In the current study, an anti-proliferation property of miR-602 was revealed, we also identified that IL-6R was turned downed by miR-602 and induced by S-3′UTR. These indicate that this miRNA might play the same biological functions in different cells. Overall, this study demonstrated that S-3′UTR of SARS-CoV-2 could bind to different miRNAs and alter the expression levels of these miRNAs in different cells (293T and HUVECs), leading to disturbances in cellular activity and various pathophysiological processes of infected cells.

mRNA vaccines are an effective prophylaxis against SARS-CoV-2 and help to prevent severe clinical presentations and poor outcomes (46, 47). RNA therapy has always been considered a promising therapeutic approach against a wide range of diseases. Insights into the roles of miRNA in disease pathogenesis have resulted in miRNAs becoming attractive targets for drug development. Mirvirasen and RG-101 are two miRNA-based medicines for acute and chronic hepatitis C, and target miR-122, which is crucial for the stability and propagation of HCV RNA (48, 49). RG-125/AZD4076, targeting miR-103/107, is undergoing clinical trials for applications in patients with type 2 diabetes and non-alcoholic fatty liver diseases (50). Moreover, MRX34, a miR-34 mimic, is used to treat multiple solid tumors (51). In the current study, the miR-296 mimic and miR-602 mimic could rescue the disturbances in cell proliferation and apoptosis induced by S-3′UTR in 293T cells and HUVECs, respectively. Therefore, development of therapeutics based on miRNAs might be an alternative and promising approach for the treatment of SARS-CoV-2 infection.

## Methods and materials

### Cell culture and transfection

293T cells and HUVECs were cultured, respectively, supplemented with 10% FBS and 1% penicillin-streptomycin (PS), at 37°C in a humidified environment with 5% CO_2_. Transfection of 293T cells and HUVECs was performed 24 h after cell seeding and used the transfection reagent Lipofectamine™ 2000 according to the manufacturer’s instructions. Briefly, 3′UTR overexpressing plasmid and miR-486 mimic were mixed with Lipofectamine™ 2000 and the mixture was added to the cell culture medium. The medium was replaced with fresh medium after 6–10 h.

### Quantitative real-time PCR and miRNA RT-PCR

Total RNA was extracted from cells with TRIzol reagent (Invitrogen, USA) following the manufacturer’s instructions, and cDNA was synthesized using the GoScript™ Reverse Transcription System (Promega) according to the manufacturer’s instructions. The qPCR reactions were performed using standard mode on a real-time PCR instrument with GoTaq^®^ qPCR Master Mix kit (Promega). Specific primers for the target miRNA and internal control were designed as: miR-1299 forward 5′-CCGCGCTTCTGGAATTCTGTGT-3′, and miR-1299 reverse 5′-AGTGCAGGGTCCGAGGTATT-3′; miR-214 forward, and miR-214 reverse; miR-296 forward 5′-CGAATATGAGGGTTGGGTGGAGG-3′, and miR-296 reverse 5′-AGTGCAGGGTCCGAGGTATT-3′; miR -302c forward, and miR -302c reverse; miR-520h forward 5′-CCGCGACAAAGTGCTTCCCTT-3′, and miR-520h reverse 5′-AGTGCAGGGTCCGAGGTATT-3′; miR-602 forward 5′-AATGACACGGGCGACAGCTG-3′, and miR-602 reverse 5′-AGTGCAGGGTCCGAGGTATT-3′; miR-766 forward 5′-CGAATACTCCAGCCCCACAGC-3′, and miR-766 reverse 5′-AGTGCAGGGTCCGAGGTATT-3′; and U6 forward, and U6 reverse. Expression levels were normalized to internal controls (U6) and results were shown in form of relative expression calculated by the 2−ΔΔCT method.

### Adenoviral shuttle plasmid construction

Plasmid pDC316 (Microbix Biosystems Inc., Toronto, ON, Canada) is an E1 shuttle plasmid derived from the left end of the adenovirus type 5 (Ad5) genome. The shuttle vector pDC316-mCMV-EGFP and antisense fragment of the 3′UTR gene of SARS-CoV-2 were restriction digested with NotI and EcoRV, respectively. The digested products were purified and ligated with T4 DNA ligase, and then co-transformed into *Escherichia coli* DH-5α. Thus, the fragment of the 3′UTR gene was cloned into the shuttle plasmid pDC316-mCMV-EGFP, and the homologous recombinant adenoviral plasmid was generated. The resulting pDC316-3′UTR-mCMV-EGFP plasmid was restriction digested with NotI and EcoRV to validate successful construction.

### Cell proliferation assay

Cell Counting Kit‐8 (CCK‐8, Dojindo, Tokyo, Japan) was used to detect cell proliferation according to the manufacturer’s instructions. Cells (approximately 2,000 per well) were seeded into 96‐well plates and cultured as previously described. Ten microliters of CCK-8 solution mixed with serum‐free medium were added every 24 h. After incubation for 2 h, the absorbance of the wells was detected using a microplate reader at a test wavelength of 450 nm.

### Apoptosis assays

The apoptosis rate was evaluated using an Annexin V-FITC/PI Apoptosis Detection kit according to the instructions from the manufacturer. Cells were seeded in 6-well tissue culture plates at 4×10^5^ cells/well. Following treatment, the cells were collected, washed with PBS, and resuspended in 500 μL binding buffer. Next, 5 μL Annexin V-FITC and 5 μL propidium iodide (PI) were added to the cell mixture and incubated at room temperature for 15 min in the dark. Cells were analyzed by flow cytometry (BD FACSCanto) within 1 h.

### Cell-cycle analysis

Cells were seeded in a 6-well tissue culture plate at 4×10^5^ cells/well. After treatment, the cells were collected, washed twice with PBS, and then 100 μL RNase A solution was added and the cells were incubated for 30 min at 37°C. Finally, 500 μL PI was added and incubated for 15 min at room temperature. The cell cycle was analyzed by flow cytometry, with data detection by Cell Quest software (Becton Dickinson, Franklin Lakes, NJ, USA). The percentage of cells in the G1, S, and G2 phases of the cell cycle were analyzed.

### Enzyme-linked immunosorbent assay

Total proteins were extracted from the cells and ELISA kits were employed to analyze the levels of TNF-α and IL-6 following the manufacturer’s instructions (Sangon Biotch, China). The absorbance at 450 nm was detected using a Power Wave Microplate Reader.

### Dual-luciferase reporter assay

The 3′-UTR of the SARS-CoV-2 fragment containing the putative binding site of miRNAs was amplified and cloned downstream of the luciferase gene in the pmirGlo vector (Sangon Biotch, China). The mutant 3′-UTR was used to construct the 3′-UTR-MUT plasmids. 293T cells, at 70–80% confluency, were co-transfected with miRNA (miR-1299, miR-214, miR-296, miR -302c, miR-520h, miR-602, and miR-766) mimics and 3′-UTR-WT. Luciferase activities were assessed 48 h post-transfection using the Dual-Luciferase Reporter Assay System (Promega Biotech Co., Madison, USA).

### Statistical analysis

All data in this study are shown as mean ± standard deviation. Statistical significance was analyzed using a two-tailed Student’s t-test to compare differences between two groups or using one-way analysis of variance (ANOVA; SPSS version 24.0, Chicago, IL, USA) to compare data among groups when they had a normal distribution and homogeneous variances. A p-value < 0.05 was considered statistically significant; *p < 0.05, **p < 0.01, ***p < 0.001.

## Data availability statement

The original contributions presented in the study are included in the article/**Supplementary Material**. Further inquiries can be directed to the corresponding authors.

## Author contributions

XH, LX, and HY designed the experiment, analyzed data and wrote the manuscript. JY, ZF, QW, and LH did almost molecular experiments and data analysis. ZF did bioinformatics. SG and LL did data analysis and helped experiment design. All authors contributed to the article and approved the submitted version.

## Funding

This work was supported by Joint Research Project of Health and Education in Fujian Province (2019-WJ-15); Fuzhou Health Science and technology innovation platform construction project (2020-S-wp5); National Natural Science Foundation of China (82172275).

## Conflict of interest

The authors declare that the research was conducted in the absence of any commercial or financial relationships that could be construed as a potential conflict of interest.

## Publisher’s note

All claims expressed in this article are solely those of the authors and do not necessarily represent those of their affiliated organizations, or those of the publisher, the editors and the reviewers. Any product that may be evaluated in this article, or claim that may be made by its manufacturer, is not guaranteed or endorsed by the publisher.
